# Varicella–Zoster Virus Reactivation Presenting as Cranial Neuritis Without Rash in Late Pregnancy: A Case Report

**DOI:** 10.1155/crog/5236883

**Published:** 2026-04-24

**Authors:** Renata Košir Pogačnik, Ana Kofol, Tatjana Mrvič, Maja Dolanc Merc

**Affiliations:** ^1^ Department of Perinatology, University Medical Centre Ljubljana, Ljubljana, Slovenia, kclj.si; ^2^ Faculty of Medicine, University of Ljubljana, Ljubljana, Slovenia, uni-lj.si; ^3^ Clinic for Infectious Diseases and Fever Conditions, University Medical Centre Ljubljana, Ljubljana, Slovenia, kclj.si

**Keywords:** case report, cranial neuritis, facial nerve palsy, pregnancy, varicella–zoster virus, zoster sine herpete

## Abstract

**Introduction:**

Neurological complications of reactivated varicella–zoster virus (VZV) are uncommon in immunocompetent individuals and are rarely reported during pregnancy. VZV reactivation may cause cranial neuritis, including Ramsay Hunt syndrome, or may present without cutaneous lesions as zoster sine herpete, complicating diagnosis.

**Case Presentation:**

We report the case of a 26‐year‐old primigravida at 37 weeks of gestation who presented with paresthesia of the left side of the tongue, dizziness, and subsequent left‐sided facial nerve palsy. Brain magnetic resonance imaging showed no abnormalities. Audiometry demonstrated mild high‐frequency hearing loss on the left side. Cerebrospinal fluid analysis revealed lymphocytic pleocytosis, and polymerase chain reaction detected VZV DNA. The patient received intravenous acyclovir (10 mg/kg every 8 h for 10 days). No vesicular rash developed during the clinical course. The clinical presentation was consistent with VZV‐associated cranial neuritis involving Cranial Nerves VII and VIII within the spectrum of zoster sine herpete. Intravenous acyclovir therapy was administered for 10 days, and corticosteroids were discontinued after virological confirmation. Delivery was performed by planned cesarean section due to breech presentation, and the newborn was healthy with no signs of neonatal varicella infection. At hospital discharge, the mother had persistent House–Brackmann grade V facial paralysis. One month after symptom onset, the facial paresis had completely resolved.

**Conclusion:**

VZV reactivation in pregnancy may present as isolated cranial neuritis without rash. Cerebrospinal fluid PCR is essential for diagnosis in atypical cases. Early antiviral treatment is associated with favorable maternal and fetal outcomes. Only a few cases of VZV meningitis or encephalitis in pregnant patients have been reported in the literature. Although immunodeficiencies are usually associated with these clinical manifestations, pregnancy is a condition in which various changes occur in the woman′s immune system. This case highlights that VZV reactivation may present as cranial neuropathy without rash during pregnancy. Detection of VZV DNA in cerebrospinal fluid is essential for diagnosis in atypical presentations. Early recognition and antiviral therapy are important for maternal neurological outcomes and are safe for the fetus.

## 1. Introduction

Varicella–zoster virus (VZV) is a neurotropic human herpesvirus of the alphaherpesvirus family that causes primary infection as varicella and establishes lifelong latency in sensory ganglia. [1] Reactivation of latent virus later in life leads to herpes zoster and may result in a wide spectrum of neurological complications. [2] Neurological manifestations associated with VZV reactivation include meningitis, encephalitis, vasculopathy, myelitis, and cranial nerve involvement. [3] Central nervous system (CNS) involvement is increasingly recognized even in immunocompetent individuals and may occur with or without the typical dermatomal rash. [4] Cranial neuritis due to VZV reactivation most commonly affects the facial nerve and may present as Ramsay Hunt syndrome. [5] The syndrome typically presents with peripheral facial nerve palsy, otalgia, and vesicular eruptions in the external auditory canal or auricle. [6] In some cases, reactivation occurs without a characteristic vesicular rash, a condition referred to as zoster sine herpete, which poses a diagnostic challenge. [7] Pregnancy is a unique immunological state characterized by complex adaptations of the maternal immune system that may alter susceptibility to viral infections. [8] Reactivation of VZV during pregnancy is uncommon but can lead to significant maternal morbidity and diagnostic challenges.[9] Although VZV‐associated CNS involvement during pregnancy has been reported, it remains rare. [10] Only a limited number of cases describing CNS involvement caused by VZV during pregnancy have been documented in the literature [11–13] Here, we present a rare case of VZV‐associated cranial neuritis in late pregnancy without rash, confirmed by cerebrospinal fluid (CSF) polymerase chain reaction (PCR), highlighting diagnostic and therapeutic considerations.

## 2. Case Presentation

A 26‐year‐old primigravida at 37 weeks of gestation presented to the neurological emergency department with neurological symptoms that had developed over several days. The condition initially began with pain in the left ear and an altered sensation on the left side of the tongue. After approximately 2 days of paresthesia, the patient developed twitching of the left eyelid, followed by dizziness and fatigue. At the time of presentation, the sensory disturbance had been present for 5 days, whereas eyelid twitching had appeared 2 days earlier. The patient had no previous neurological disorders and no chronic medical conditions. She reported a history of uncomplicated chickenpox during early primary school. Her pregnancy had been uncomplicated, and the only medication she was taking was oral iron supplementation for anemia.

### 2.1. Initial Neurological Examination

On examination, the patient was unaffected, eupnoic, anicteric, acyanotic, afebrile (36.3°C), normotensive (120/70 mmHg), with a pulse of 118/min and oxygen saturation of 97%. She was alert and oriented, with normal speech. Cranial nerve examination was initially unremarkable, and no meningeal signs were present. Examination of the upper limbs revealed no abnormalities. Examination of the lower limbs revealed increased muscle tone and hyperreflexia on the left side, with transient ankle clonus and an extensor plantar response. As these findings suggested possible upper motor neuron involvement, the neurological examination was repeated the following day by a senior neurologist. At that time, these signs were no longer reproducible and were therefore interpreted as transient and likely nonpathological.

### 2.2. Neuroimaging

Magnetic resonance imaging (MRI) of the brain was performed using a multiple sclerosis protocol. Imaging revealed a very small punctate FLAIR hyperintensity in the subcortical white matter of the left frontal lobe above the insula. This finding was considered nonspecific and possibly represented a slightly widened perivascular space. No demyelinating lesions, ischemic changes, or brainstem abnormalities were observed. The CSF spaces were of normal width. The pituitary gland appeared somewhat prominent with a convex superior margin, a finding considered physiological during pregnancy. The orbits and paranasal sinuses were within normal limits. Overall, the MRI examination was interpreted as within normal limits. Given normalization of neurological findings, spinal MRI and internal auditory canal imaging were not pursued.

### 2.3. Clinical Progression

The following day, the patient developed acute left‐sided facial paralysis. Neurological examination confirmed peripheral facial nerve palsy, and treatment with oral methylprednisolone was initiated. As the facial paralysis was accompanied by ipsilateral ear pain, the patient was referred for otorhinolaryngological evaluation. No vesicular lesions were observed in the external auditory canal or elsewhere on the skin. Audiometric testing demonstrated mild high‐frequency hearing impairment on the left side (approximately 35 dB), whereas hearing on the right side was normal. A video head impulse test (vHIT) was also performed. The examination demonstrated vestibular dysfunction on the left side, indicating involvement of the vestibular nerve. These findings suggested that, in addition to the facial nerve (Cranial Nerve VII), the vestibulocochlear nerve (Cranial Nerve VIII) was also affected. Based on these findings, the patient was referred to the Clinic for Infectious Diseases (KIBVS) for further diagnostic evaluation of a possible CNS infection with neurotropic viruses. At this stage, the severity of the facial nerve dysfunction was clinically assessed as Grade V according to the House–Brackmann scale, indicating severe facial nerve impairment (Table 1).

**Table 1:** Clinical timeline of VZV reactivation in pregnancy.

Timeline	Day −5	Day −3	Day 0	Day +1	Day +2	Day +10	1 month	6 months
Symptoms onset	Tongue paraesthesia	Periorbital twitching	Dizziness, fatigue	Facial palsy develops	Otalgia, dizziness, audiometry	—	Complete facial recovery	Stable, no recurrence
Neurological exam	—	Initial UMN signs	Repeat exam: normalized	Peripheral facial weakness	—	—	Normal	Normal
Imaging	—	Brain MRI normal	—	—	—	—	—	—
CSF	—	Lumbar puncture performed	CSF Lymphocytic pleocytosis; VZV PCR positive	—	—	—	—	—
Treatment	—	—	Oral steroid initiated	—	Corticosteroids discontinued; IV acyclovir initiated	10‐day IV acyclovir completed	Ocular care/facial physiotherapy	—
Obstetric course	—	—	Fetal monitoring	—	—	Planned cesarean section	—	—
Neonatal outcome	—	—	—	—	—	Healthy neonate	—	—

*Note:* Legend: IV acyclovir = intravenous antiviral therapy.

Abbreviations: CSF, cerebrospinal fluid; UMN, upper motor neuron signs.

### 2.4. Infectious Work‐Up

The patient underwent further evaluation at an infectious diseases clinic to investigate possible infectious causes of cranial nerve involvement, including Lyme disease and neurotropic viral infections. She reported no history of tick bites and had been vaccinated against tick‐borne encephalitis. Laboratory tests showed mildly elevated leukocytes at 13.7 (reference range: 4.0–10.0). Serological testing for *Borrelia burgdorferi* and tick‐borne encephalitis virus was negative.

### 2.5. CSF Analysis

A lumbar puncture was performed. The CSF was clear and transparent. CSF analysis showed 22 leukocytes/*μ*L, erythrocytes fewer than 500/*μ*L, protein 0.35 g/L (reference range: 0.15–0.45), and glucose 2.9 mmol/L (reference range: 2.5–3.9). Microscopic examination revealed a cellular pattern predominantly of small monomorphic lymphocytes with scant cytoplasm and oval nuclei containing granular chromatin. Monocytes, plasma cells, and occasional naked nuclei were also observed. Flow cytometric immunophenotyping showed 89% CD3‐positive T lymphocytes, 7.6% CD56‐positive natural killer cells, 0.3% CD19/CD20‐positive polytypic B cells, and 0.4% CD14‐positive monocytes. The CD4/CD8 T‐lymphocyte ratio was 1.5:1, and no malignant cells were detected. These findings were consistent with lymphocytic pleocytosis. PCR testing of the CSF detected VZV DNA, whereas tests for herpes simplex virus Types 1 and 2 were negative.

### 2.6. Diagnosis and Treatment

Based on the clinical presentation and CSF findings, the patient was diagnosed with VZV‐associated cranial neuritis involving the facial nerve (VII) and vestibulocochlear nerve (VIII) within the spectrum of zoster sine herpete. Intravenous acyclovir (10 mg/kg every 8 h) was initiated and continued for 10 days. Corticosteroid therapy with methylprednisolone was discontinued after confirmation of the viral infection. The patient also underwent regular physiotherapy for facial nerve rehabilitation, including ocular lubrication, nighttime eye patching, and facial physiotherapy.

### 2.7. Obstetric Course

Continuous fetal cardiotocographic monitoring during hospitalization showed reassuring findings. As the fetus was in breech presentation, a planned cesarean section was performed. A healthy neonate was delivered weighing 3060 g and measuring 48 cm, with Apgar scores of 9 at 1 and 5 min. The neonate required only routine postnatal observation and exhibited no signs of VZV infection. Breastfeeding was allowed. At discharge, the patient′s neurological symptoms, particularly the left‐sided facial palsy, remained and corresponded to Grade V facial paralysis on the House–Brackmann scale.

### 2.8. Follow‐Up

After starting antiviral therapy and continuing physiotherapy, the patient began to notice clinical improvement about 4 days after treatment began. Over the following weeks, gradual recovery of facial nerve function occurred, and about 1 month after symptom onset, the facial paresis had completely resolved. At 6‐month follow‐up, the patient remained asymptomatic with no recurrence (Table 2).

**Table 2:** Serial neurological examination (day‐by‐day).

Day	Time	Cranial nerves	Upper limb	Lower limb	Reflexes	Tone	Plantar response	Comments
0 (admission)	Morning	Normal	Normal	Mild left hypertonia	Normal	Mild increased on left	Extensor (left)	Initial UMN signs noted
0	Evening	Normal	Normal	Normal	Normal	Normal	Flexor	Repeat exam by senior neurologist; UMN signs not reproducible
1	Morning	Normal	Normal	Normal	Normal	Normal	Flexor	Confirmed normalization
2	Morning	Left facial weakness (VII)	Normal	Normal	Normal	Normal	Flexor	Peripheral facial palsy developed; UMN signs absent
1 month	—	Normal	Normal	Normal	Normal	Normal	Flexor	Complete recovery of facial nerve function
6 months postpartum	—	Normal	Normal	Normal	Normal	Normal	Flexor	Stable, no recurrence

*Note:* Legend: Findings confirm transient, nonreproducible UMN signs; final diagnosis focused on cranial neuritis.

Abbreviation: UMN, upper motor neuron.

## 3. Discussion

Several case reports of neurological complications associated with VZV reactivation during pregnancy have been published, although the number remains very small. Previously reported cases include VZV meningitis or encephalitis in women with comorbidities such as diabetes mellitus or human immunodeficiency virus infection. [10–14] Additional reports describe cases in otherwise healthy pregnant individuals, highlighting the unpredictable nature of the disease. [11–13] Table 3 summarizes previously published cases with CNS involvement during pregnancy, including maternal age, gestational age at presentation, underlying comorbidities, diagnostic methods, treatment administered, and clinical outcomes. Comparison with earlier reports highlights the rarity of this condition and the limited evidence available to guide management.

**Table 3:** Reported cases of varicella‐zoster virus meningitis during pregnancy in the literature.

Author	Year	Gestation	Rash	Immune Status	Clinical presentation	Treatment	Neonatal Outcome	DOI
Jayakrishnan et al.	2008	3rd trimester	Yes	HIV	Meningitis	IV acyclovir	Healthy neonate	10.1055/s‐0028‐1085625
Mroue et al.	2021	25 weeks	No	Type 1 DM	Meningitis	IV acyclovir	Term delivery, healthy	10.1136/bcr‐2020‐236644
Gholkar and Verghese	2020	32 weeks	No	Immunocompetent	Meningitis	IV acyclovir	Healthy neonate	10.18203/2320‐1770.ijrcog20200921
Troup and Berkman	2025	2nd trimester	Yes	Immunocompetent	Meningitis	IV acyclovir	Favorable	10.1212/WNL.0000000000208671
**Present case**	2026	37 weeks	No	Immunocompetent	Cranial neuritis (VII, VIII)–zoster sine herpete	IV acyclovir	Healthy neonate	—

In contrast to previously reported cases primarily categorized as meningitis, the present case is more accurately defined as VZV‐associated cranial neuritis, based on the clinical presentation. The patient exhibited focal neurological deficits involving the facial (VII) and vestibulocochlear (VIII) nerves, without accompanying headache, fever, photophobia, or meningeal signs. Although CSF analysis demonstrated lymphocytic pleocytosis and confirmed the presence of VZV DNA, these findings likely reflect limited viral spread rather than clinically overt meningitis. This distinction is clinically important, as CSF abnormalities may occur in VZV reactivation even in the absence of true meningeal involvement. The key clinical differences between VZV cranial neuritis and VZV meningitis are summarized in Table 4.

**Table 4:** VZV cranial neuritis versus VZV meningitis.

Feature	VZV cranial neuritis	VZV meningitis
Anatomical involvement	Single cranial nerve (most commonly VII, VIII, and V)	Meninges
Pathogenesis	Reactivation of VZV in a ganglion (e.g., geniculate ganglion)	Hematogenous or perineural spread to the meninges
Typical clinical presentation	Focal deficit (e.g., facial nerve palsy, pain, and vertigo)	Headache, fever, photophobia, and nausea
Skin rash	Common (herpes zoster oticus, and herpes zoster faciei)	May be present or absent
Systemic signs	Usually mild or absent	Common
Meningeal signs	No	Often
Disturbance of consciousness	No	Possible
CSF (lumbar puncture)	Often normal or mildly altered	Lymphocytic pleocytosis, ↑ protein
Life‐threatening	Usually not	Can be (especially in immunosuppressed patients)
Prognosis	Mostly good, possible sequelae	Usually good, complications are rare

VZV‐related neurological disease associated with primary or secondary infection may present without a vesicular rash and with only focal neurological symptoms in up to 55% of cases, as some studies suggest. [15–17] The absence of cutaneous lesions does not exclude VZV reactivation. Previous studies suggest that a proportion of VZV‐related facial nerve palsies occur without vesicular eruptions. In such cases, laboratory confirmation through PCR testing of CSF is often necessary to establish the diagnosis. [18, 19] The absence of dermatological findings in our patient underscores the importance of considering VZV in the differential diagnosis of acute cranial neuropathies, even in immunocompetent pregnant individuals. Similar neurological progression has been described in rare cases outside pregnancy. [20] Ramsay Hunt syndrome results from viral reactivation in the geniculate ganglion and may involve multiple cranial nerves. [5] Treatment typically involves prompt initiation of antiviral therapy combined with corticosteroids. Evidence indicates that combination therapy improves facial nerve recovery compared with antiviral therapy alone. [21] Early treatment is therefore recommended to reduce the risk of persistent neurological deficits. Antiviral agents such as acyclovir are generally considered safe during pregnancy. Large epidemiological studies have not shown an increased risk of major congenital malformations following first‐trimester exposure to acyclovir or related antiviral medications. [22] Additionally, antiviral therapy is widely recommended for the management of maternal VZV infection when clinically indicated. [23] Recent reviews emphasize that herpes zoster during pregnancy rarely leads to fetal complications but may cause significant maternal morbidity if neurological complications occur. [24, 25] Early recognition and treatment are therefore essential to optimize maternal outcomes. This case highlights that VZV reactivation may present as isolated cranial neuritis without rash, and that CSF findings alone should not lead to overclassification as meningitis in the absence of corresponding clinical features. Prompt diagnostic evaluation and early antiviral therapy are essential for preventing complications and improving clinical outcomes.

## 4. Conclusion

VZV reactivation should be considered in pregnant patients presenting with cranial nerve deficits, even in the absence of rash.

This case supports cranial neuritis as the primary clinical entity, with CSF findings representing secondary involvement. Prompt diagnostic evaluation and early antiviral therapy are essential to prevent complications and improve clinical outcomes.

## Author Contributions

All authors contributed substantially to the conception and design of the study, acquisition of clinical data, and drafting of the manuscript. All authors critically revised the manuscript for important intellectual content. **Renata Košir Pogačnik:** conceptualization, data collection, writing – original draft, writing – review and editing. **Maja Dolanc Merc**: conceptualization, clinical management, data collection, writing – original draft, writing – review and editing. **Tatjana Mrvič:** clinical management, data collection, writing – review and editing. **Ana Kofol:** writing – original draft, writing – review and editing.

## Funding

The authors received no external funding for this work.

## Disclosure

All authors approved the final version for publication.

## Ethics Statement

Written informed consent for publication of this case report and accompanying clinical information was obtained from the patient. Institutional ethical approval was not required for a single anonymized case report according to local regulations.

## Consent

Written informed consent was obtained from the patient for publication of this case report. We use our consent to publication form, and we provide a blank copy of the consent form on submission.

## Conflicts of Interest

The authors declare no conflicts of interest.

## Data Availability

The data that support the findings of this study are available from the corresponding author upon reasonable request.
